# Successful Management of Total Knee Replacement in a High Responder Hemophilia Patient With a History of Inhibitor

**DOI:** 10.5812/ircmj.3406

**Published:** 2013-01-05

**Authors:** Roya Dolatkhah, Mohammad Reza Bazavar, Masoud Poureisa, Iraj Asvadi Kermani, Jalil Vaez Gharamaleki, Zohreh Sanaat, Jamal Eivazi Ziaei, Alireza Nikanfar, Ali Esfahani, Seyed Hadi Chavoshi

**Affiliations:** 1 Hematology and Oncology Research Center, Tabriz University of Medical Sciences, Hemophilia and Thalassemia Department, Tabriz, IR Iran; 2 Department of Orthopedic, Tabriz University of Medical Sciences, Tabriz, IR Iran; 3 Radiology Department, Tabriz University of Medical Sciences, Tabriz, IR Iran

**Keywords:** Orthopedics, Hemophilia A, Hemophilia B, Inhibitor

## Abstract

The development of inhibitors against administered clotting factors may render replacement therapy ineffective for some hemophilia patients. Such patients are therefore at the highest risk of developing arthropathy. Elective orthopedic surgery (EOS) in hemophilic patients having such inhibitors remains a rare, expensive, and difficult surgery, whose management represents a significant challenge. We report the case of a 35-year-old man with a severe form of hemophilia A (factor VIII < 1%), who was suffering from repetitive spontaneous hemarthrosis, especially in his knee joints that had consequently become more susceptible to bleeding. The patient had a history of high levels of factor VIII inhibitor (> 5.0 Bethesda Unit [BU]/ml) as shown by the factor VIII inhibitor assay; therefore, we began treatment with factor VIIa for his mild-to-moderate bleeding (90 µg/kg intravenous bolus injections). The interval between injections varied with the severity of the hemorrhage in each bleeding episode. The inhibitor level reduced to 3.1 BU/ml after three months, to 1.6 BU/ml after six months, and disappeared completely after one year of treatment. We administered factor VIII at a dose of 50 IU/kg every eight hours during the first three post-operative days, then continued administration with a dose of 40 IU/kg every 12 hours for another four days, and observed a very good response to treatment with no bleeding. Recombinant activated factor VII (rFVIIa) is not an inhibitor-removal strategy, but an inhibitor-bypassing product. However, in our patient, the treatment of mild-to-moderate bleeding with short-term use of rFVIIa and no exposure to factor VIII caused a gradual reduction in the inhibitor level over a period of 1 year.

## 1. Introduction

Hemophilic arthropathy is caused by recurrent episodes of hemorrhage into the joint, and if left untreated, it can lead to severe chronic pain and permanent functional disability (1). The most commonly affected joints are the knee, ankle, and elbow. However, the development of inhibitors against administered clotting factors in some hemophilia patients may render replacement therapy ineffective, and these patients are therefore at the highest risk of developing arthropathy (1). Although clotting factors are known to be effective for the treatment of arthropathy, joint bleeding and hence blood-induced joint damage are still commonly observed. There are several reasons for this, one of which is that not all patients (whether in our center or worldwide) have access to sufficient amounts of clotting factor, either due to limited availability or high costs. Although the number of cases and severity of joint bleeding reduces with the administration of clotting factors, such bleeding still occurs in a significant number of cases (2). Elective orthopedic surgery (EOS) is a last resort for patients with progressive arthropathy and orthopedic complications and end-stage arthropathy of most large joints (3, 4). It can provide long-term cost savings as it reduces the bleeding frequency and causes a significant drop in pain levels and moreover restore mobility and function with minimal joint damage (4, 5). Additionally by and large, favorable functional outcomes following total knee replacement in hemophilic patients have been reported, with a favorable improvement in the clinical scores and range of movements (ROM) of patients (4). Nevertheless, approximately 20%–25% of hemophilia A patients and 1%–3% of hemophilia B patients have inhibitors for factor VIII and factor IX. Such cases represent a major therapeutic challenge to clinicians (5). Arthroplastic surgery in hemophilic patients having such inhibitors remains a rare, expensive, and difficult surgery, whose management represents a significant challenge (6). Since 1988, recombinant activated factor VII (rFVIIa) have been indicated for use in surgical prophylaxis, as well as for the treatment of bleeding episodes, in patients with hemophilia and high-responding inhibitors. Many studies support the use of rFVIIa as a first-line therapy in surgery for hemophilia patients with high-responding inhibitors, as it enables a safe surgical procedure without causing anamnesis (7, 8).

## 2. Case Report

We report the case of a 35-year-old man with a severe form of hemophilia A (factor VIII < 1%), who was suffering from repetitive spontaneous hemarthrosis, principally in his knee joints that had consequently become more susceptible to bleeding (Target Joint). The patient was diagnosed and treated at our center in 1993. He was first treated with cryoprecipitate, because we did not have factor concentrates at our center. Since 1996, he received factor concentrates (on demand) however, the amounts were insufficient. In addition, he did not receive any prophylaxis treatment. During the last year, repetitive spontaneous hemarthrosis led to chronic synovitis and ultimately to degenerative arthritis, and his arthropathy became more severe. Because of repeated hemarthrosis, he developed synovial hypertrophy, cartilage destruction, bone damage, and disabling arthritis, which is known as chronic hemophilic arthropathy and impairs joint function and inducing pain. The patient was disabled due to persistent pain and dysfunction caused by osteoarthritis secondary to progressive severe hemophilic arthropathy; therefore, the patient was referred to our Orthopedic Consultant. Upon physical examination of both knees, the following findings were noted:

1. Pain-reduced ROM and deformities

2. Grade IV arthropathy in both knees

3. ROM of the knees was 0–90° without contracture (8)

**Figure 1:**
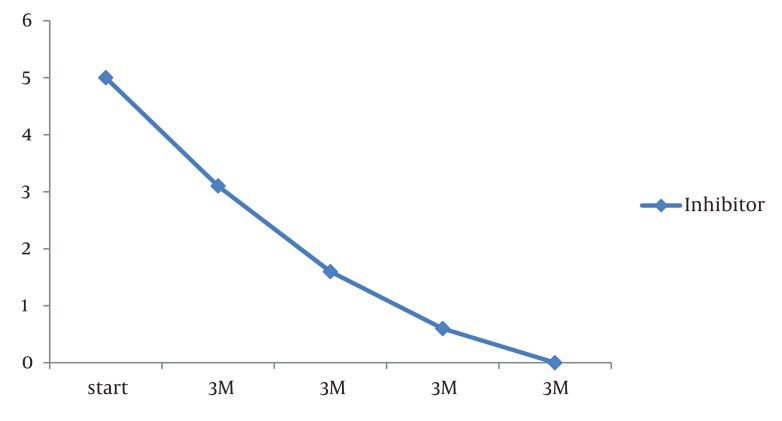
The Inhibitor Assay of Patient During Treatment

Radiological assessment revealed enlargement of the epiphysis, osteoporosis, erosions, osteophyte formation, cartilage damage, and ankylosis. Hemarthrosis led to inflammatory changes in the synovial tissue and degenerative changes in the cartilage as well as affecting the subchondral bone. The patient had tested positive for Hepatitis C Virus, which was treated seven years earlier with interferon and ribavirin, and this treatment showed a favorable response. He also had a history of high levels of factor VIII inhibitor (> 5.0 Bethesda Unit [BU]/ml) as shown by the factor VIII inhibitor assay. We therefore began treatment with factor VIIa via 90 µg/kg intravenous bolus injection for his mild-to-moderate bleeding. The interval between injections varied with the severity of the hemorrhage in each bleeding episode. The inhibitor level reduced to 3.1 BU/ml after three months, to 1.6 BU/ml after 6 months, with complete remission within one year of treatment (Figure 1).

In a recent radiograph of the patient’s left knee, we found lateral osteophyte formation in the lateral tibia, fusion of the patella in the distal end of the femur, and a severe reduction in the joint space. The patient was in the care of a multidisciplinary team of specialists including an orthopedic surgeon, a hematologist, and a physiotherapist. Total knee arthroplasty (TKA) was then performed on his right knee. We used factor VIII at a dose of 50 IU/kg every eight hours during the first three post-operative days, subsequently continued its administration at a dose of 40 IU/kg every 12 hours for another four days, and observed a very good response to treatment with no bleeding.

## 3. Discussion

The interesting feature of this case is that the inhibitor titer of a patient with hemophilia was successfully reduced from > 5.0 BU/ml to approximately 0 BU/ml within one year, without surgery, suggesting that the patient did not require any high-dose or high-costing bypassing agents during the TKA operation. A 90 µg/kg dose of rFVIIa has been shown to provide effective hemostasis in over 90% of mild-to-moderate bleeding cases after a mean of 2.2 injections (9). Hence, only mild-to-moderate bleeding can be controlled with a low dose (and low cost) of rFVIIa, and the inhibitor level reduced in our patient because he was not exposed to factor VIII. According to Soren et al., the efficacy of on-demand treatment of spontaneous bleeding in inhibitor patients can be optimized by prescribing the highest appropriate dose of rFVIIa based on patients' previous response history. Due to an excellent response of our patient to a 90 µg/kg dose of rFVIIa, we did not consider the initially recommended dose of 270 µg/kg (9). Solimeno et al. reported that a high responder patient had a 1 BU/ml antibody titer at the time of surgery; a high dose of factor VIII (52000 U) was used for the first five days and on the fifth day after surgery, rFVIIa was administered for two days (6). In our case, the patient’s inhibitor titer was below 1 BU/ml before surgery, and we controlled his bleeding during and after surgery with a high dose of FVIII. Administration of bypassing agents was therefore unnecessary because of the excellent response to factor VIII. In the first randomized clinical trial reported to date on the prophylaxis in hemophilia patients having such inhibitors, rFVIIa reduced the bleeding frequency, duration of hospital stay, and duration of absence from work/school and improved the quality of life as compared to conventional on-demand therapy. In a pharmaco-economic analysis of bypassing agents in the treatment of minor-to-moderate bleeding in hemophilia patients with such inhibitors, the rFVIIa-only strategy was the least expensive (10). It is clear that rFVIIa is not an inhibitor-removal strategy; instead, it is an inhibitor-bypassing product. However, in our case, because of the short-term use of rFVIIa for the treatment of mild-to-moderate bleeding and because the patient was not exposed to factor VIII, a gradual reduction of inhibitor level was possible over a period of one year. It is recommended that only in long-term treatments, immune tolerance may be induced, and desensitizing the immune system and eradicating the inhibitor by the infusion of frequent large doses of factor replacement may be possible. However, short-term, cost effective, and safe solutions may also be required, particularly during the management of acute bleeding episodes (11). EOS in hemophilic patients is a complex procedure that should be performed in a tertiary center with support from a dedicated hemophilia team. Further trials on such patients are needed to confirm our findings.
